# Energy Conservation in the Acetogenic Bacterium *Clostridium aceticum*

**DOI:** 10.3390/microorganisms9020258

**Published:** 2021-01-27

**Authors:** Anja Wiechmann, Volker Müller

**Affiliations:** Molecular Microbiology and Bioenergetics, Institute of Molecular Biosciences, Johann Wolfgang Goethe University Frankfurt, Max-von-Laue-Str. 9, 60438 Frankfurt am Main, Germany; a.wiechmann@bio.uni-frankfurt.de

**Keywords:** energy conservation, respiratory chain, acetogenic bacteria, Wood–Ljungdahl pathway, ATP synthase, Rnf complex

## Abstract

In times of global warming caused by the extensive use of fossil fuels, the need to capture gaseous carbon compounds is growing bigger. Several groups of microorganisms can fix the greenhouse gas CO_2_. Out of these, acetogenic bacteria are role models in their ability to reduce CO_2_ with hydrogen to acetate, which makes acetogens prime candidates for genetic modification towards biotechnological production of value-added compounds from CO_2_, such as biofuels. However, growth of acetogens on gaseous substrates is strongly energy-limited, and successful metabolic engineering requires a detailed knowledge of the bioenergetics. In 1939, *Clostridium aceticum* was the first acetogen to be described. A recent genomic study revealed that this organism contains cytochromes and therefore may use a proton gradient in its respiratory chain. We have followed up these studies and will present data that *C. aceticum* does not use a H^+^ but a Na^+^ gradient for ATP synthesis, established by a Na^+^-Rnf. Experimental data and in silico analyses enabled us to propose the biochemistry and bioenergetics of acetogenesis from H_2_ + CO_2_ in *C. aceticum*.

## 1. Introduction

Acetogenic bacteria are a group of strictly anaerobic, facultative, chemolithoautotrophic bacteria [1]. During lithotrophic growth, H_2_ + CO_2_ is converted to acetate by a specialized pathway, the Wood–Ljungdahl pathway (WLP) [2,3]. Out of the known CO_2_-fixation pathways, the WLP is the only one that does not require net input of ATP [4]. The two molecules of CO_2_ that are converted to acetate are reduced in two branches [3,4,5,6]. In the carbonyl branch, one CO_2_ is reduced to enzyme-bound carbon monoxide by CO dehydrogenase/acetyl-CoA synthase (CODH/ACS) [3,7]. In the methyl branch, CO_2_ is first reduced to formate, which is bound in a reaction driven by ATP hydrolysis to the C1 carrier tetrahydrofolate (THF) and then subsequently reduced via methenyl- and methylene-THF to methyl-THF. The methyl group is then transferred by a methyltransferase to the CODH/ACS, where it condenses with enzyme-bound CO and CoA to acetyl-CoA which is further converted via acetyl-phosphate to acetate [8]. The last reaction regains the one mol of ATP invested in the second reaction and thus, the amount of ATP synthesized in this pathway is zero. Since the bacteria grow on H_2_ + CO_2_ while producing acetate, the entire lithotrophic metabolism must be coupled to additional ATP synthesis [6].

In recent years it has been shown that acetogens use the electron transfer pathway to the WLP as the site of energy conservation by a chemiosmotic mechanism [6,9]. So far, two species have been investigated in detail. *Thermoanerobacter kivui* has a reduced ferredoxin:H^+^ oxidoreductase (Ech) [9] as a respiratory enzyme, whereas *Acetobacterium woodii* has a reduced ferredoxin:NAD^+^ oxidoreductase (Rnf) as the one and only coupling site [10,11]. Both respiratory enzymes use the free energy change of electron transport to expel ions (H^+^/Na^+^) from the cytoplasm, thus establishing a transmembrane electrochemical ion gradient across the membrane that drives ATP synthesis via a membrane-bound F_1_F_O_ ATP synthase [12]. The fuel for the electron transport chain (reduced ferredoxin) is generated by electron bifurcation with hydrogen as reductant [13]. Every acetogen sequenced so far has either Rnf or Ech and at present, the presence of *ech* and *rnf* genes are mutually exclusive with a prevalence for Ech in thermophilic species [9,14]. The situation is more complicated by the finding of cytochromes in some acetogens whose role in bioenergetics is poorly understood [14,15]. Nevertheless, the presence of cytochromes is indicative of proton-based bioenergetics. In 2015, the genome of the first isolated acetogen, *Clostridium aceticum* was sequenced [14]. As well as *rnf* genes, cytochrome-encoding genes were found and therefore, *C. aceticum* was considered a missing link between Rnf and cytochrome-containing acetogens, although the same study questioned a role of cytochromes in the bioenergetics in *C. aceticum*. It was postulated, based on the genome sequence, that *C. aceticum* has a proton-based bioenergetic. We have checked this hypothesis by studying the core components of energy conservation and their ion-dependence. These studies revealed a Na^+^-dependent respiratory chain in *C. aceticum* with a Na^+^-dependent Rnf and ATP synthase.

## 2. Materials and Methods

### 2.1. Conditions for Growth of C. aceticum

*C. aceticum* (DSM1496) was grown under strictly anoxic conditions in medium described by Braun et al. (1981) and modified by Poehlein et al. (2015) [14,16]. Generally, cells were grown at 30 °C in medium containing 20 mM fructose, and a gas atmosphere that was changed from N_2_ + CO_2_ (80:20, [*v*/*v*]) to 100% H_2_ prior to inoculation, to increase the pH to around 8.4, due to the partial conversion of carbonate to CO_2_ in the liquid, which then diffuses into the headspace. For purification of the methylene-THF reductase cells were grown in a 20 L-flask (Glasgerätebau Ochs, Bovenden-Lenglern, Germany) on 20 mM fructose without the addition of H_2_.

### 2.2. Purification of Cytosol and Membranes from C. aceticum

All steps were performed at room temperature under anoxic conditions in an anaerobic chamber (Coy Laboratory Products, Grass Lake, MI, USA) filled with 96% N_2_ and 4% H_2_. For isolation of the cytosol of *C. aceticum*, cells were harvested and washed in buffer A (50 mM Tris-HCl, 20 mM MgSO_4_, 2 mM dithioerythritol (DTE), 4 µM resazurin, pH 8). To disrupt the cells, cells were resuspended in buffer A containing 0.5 mM phenylmethylsulfonyl fluoride (PMSF) and 0.1 mg/mL DNaseI, and were passed twice through a French pressure cell at 110 MPa. The same procedure was performed for isolation of membranes, with the only difference that buffer A was prepared with sodium-free chemicals. Cell debris together with membranes were then separated from the cytosolic fraction by ultracentrifugation at 130,000× *g* for 45 min. The membrane fraction was resuspended in buffer A, and as with the cytosolic fraction, it was stored in an anoxic tube at 4 °C until use.

### 2.3. Purification of the Methylene-Tetrahydrofolate Reductase

77 mL of the cytosolic fraction (60 mg/mL protein) from a 20 L culture were applied to a Q-Sepharose high-performance (HP) column (GE Healthcare, Chicago, IL, USA) equilibrated with buffer 1 (50 mM Tris-HCl, 20 mM MgSO_4_, 20% glycerol, 2 mM DTE, 4 µM resazurin, pH 7.6) using a flow rate of 2 mL/min. Protein was eluted with a linear gradient of 150 mL from 0 to 500 mM NaCl in buffer 1. Methylene-THF-dependent oxidation of reduced methyl viologen was detected in the eluate containing around 150–220 mM NaCl. Ammonium sulfate (2.4 M) was added to the pooled fractions (13 mL, 15.5 mg/mL protein). The pool was loaded onto a Phenyl-Sepharose HP column (GE Healthcare, Chicago, IL, USA), equilibrated with buffer 1 containing 2.4 M (NH_4_)_2_SO_4_ under a flow rate of 1.3 mL/min. Protein was eluted with a linear gradient of 200 mL from 2.4 to 0.0 M (NH_4_)_2_SO_4_. Methylene-THF-dependent oxidation of reduced methyl viologen eluted in a peak of around 480–160 mM remaining (NH_4_)_2_SO_4_. Pooled fractions were concentrated using ultrafiltration in 100-kDa Vivaspin tubes (Sartorius Stedim Biotech GmbH, Germany). The concentrated sample was separated on a HiPrep Sephacryl S-300 HR column (GE Healthcare, Chicago, IL, USA) equilibrated with buffer 1 containing 250 mM NaCl using a flow rate of 0.5 mL/min. Activity was found in fractions, which eluted after 20–30 mL.

### 2.4. Measurement of Rnf Activity

Fd_red_:NAD^+^ oxidoreductase activity and its dependence on sodium ions was measured as described before [17]. Briefly, 1.8 mL anoxic cuvettes (Glasgerätebau Ochs, Bovenden, Germany) were filled with buffer (20 mM Tris-HCl, 2 mM DTE, 2.2 μM resazurin, pH 7.7, contaminating Na^+^ concentration 104 µM Na^+^) in an anaerobic chamber (Coy Laboratory Products, Grass Lake, MI, USA) filled with 96% N_2_ and 4% H_2_ and sealed with rubber stoppers. Different amounts of NaCl, KCl, and LiCl were added and the head space of the cuvette was changed to CO. Reduction of NAD^+^ (3 mM) was monitored at 340 nm over time after addition of ferredoxin (30 µM), purified from *Clostridium pasteurianum* [17] and 250 µg of purified membrane. Ferredoxin was reduced by CODH/ACS, isolated from *A. woodii* as described by [17].

### 2.5. Measurement of ATPase Activity

ATPase activity was measured in buffer (100 mM Tris-HCl, 100 mM maleic acid, 20 mM NaCl, 5 mM MgCl_2_, 10 µM Na^+^) at 30 °C. The pH was adjusted to 7.4 with KOH. The sample was preincubated for 3 min at 30 °C before addition of 3 mM Tris-ATP to start the reaction. ATP-dependent formation of inorganic phosphate was followed as described by Heinonen and Lahti (1981) [18]. Samples were measured photometrically at 650 nm.

### 2.6. Measurements of Methylene-Tetrahydrofolate Reductase Activity

Methylene-THF activity was measured photometrically at 604 nm under anoxic conditions in 1.8 mL anoxic cuvettes (Glasgerätebau Ochs, Bovenden, Germany) under a N_2_ atmosphere filled with buffer (50 mM KPO_4_, 5 mM MgCl_2_, 2 mM DTE, 4 μM resazurin, pH 7.5). The oxidation of 10 mM methyl viologen (prereduced with sodium dithionite) was observed over time after addition of 1.5 mM formaldehyde + 0.5 mM tetrahydrofolate (THF) (Sigma-Aldrich, St. Louis, MO, USA), resulting in a racemic mixture containing 0.25 mM methylene-THF [19,20], and protein from the respective purification step of the methylene-THF reductase.

### 2.7. Measurements of Methylene-Tetrahydrofolate Dehydrogenase Activity

Methylene-THF dehydrogenase activity was measured photometrically at 340 nm under anoxic conditions in 1.8 ml anoxic cuvettes (Glasgerätebau Ochs, Bovenden, Germany) under a N_2_ atmosphere filled with buffer (50 mM KPO_4_, 2 mM DTE, pH 7.0) [21]. The reduction of NAD^+^ was measured after addition of 1.5 mM formaldehyde + 0.5 mM THF (Sigma-Aldrich, St. Louis, MO, USA), resulting in a racemic mixture containing 0.25 mM methylene-THF, and 30 µg of cytosol from *C. aceticum*.

### 2.8. Analytical Methods

Soluble proteins were quantified using the method of Bradford (1976) [22], and membrane proteins by the method of Lowry et al. (1951) [23]. Proteins were separated in 12% SDS-polyacrylamide gels and stained with Coomassie Brilliant Blue. Native gel electrophoresis was performed as described before [24]. For genetic analyses, the Basic Local Alignment Search Tools (BLAST) from the National Center for Biotechnology Information (NCBI, Bethesda, MD, USA) was used. Sequence comparisons were performed with the Clustal Omega tool from the European Bioinformatics Institute (EMBL-EBI, Hinxton, UK). The sodium ion concentration was measured using a Na^+^ specific electrode (Orion Star A214, Thermo Scientific, Waltham MA, USA). To reduce Na^+^ contaminations, ultra-pure chemicals and buffer with low concentrations of ingredients were used.

## 3. Results

### 3.1. The Rnf Complex from C. aceticum Requires Na^+^ for Activity

**Figure 1:**
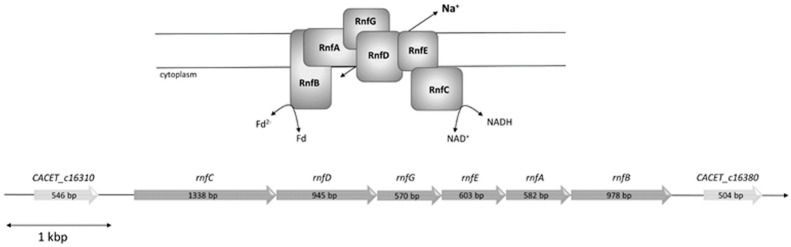
Model and genetic organization of the *rnf* operon in *C. aceticum*. The *rnf* genes are organized in a potential operon consisting of six genes, which code for a membrane-bound complex potentially consisting of monomers of each subunit. The first one is CACET_c16320 coding for RnfC, followed by CACET_c16330 coding for RnfD, CACET_c16340 coding for RnfG, CACET_c16350 coding for RnfE, CACET_c16360 coding for RnfA and CACET_c16370 coding for RnfB. The operon is flanked upstream by CACET_c16310, which has high similarity with an ATP-binding protein (sensor histidine kinase) and downstream by CACET_c16380, which has similarity with a transporter protein.

*C. aceticum* contains six genes encoding an Rnf complex (CACET_c16320-CACET_c16370), which are potentially organized in an operon (Figure 1). The order of the genes is identical to *A. woodii* and the number of nucleotides of the genes are roughly the same. The RnfB subunit can differ in length as shown for various organisms [25]; however, the RnfB of *A. woodii* and RnfB of *C. aceticum* have almost the same amount of amino acids (334 in *A. woodii* and 329 in *C. aceticum*) and show 51% homology. As in the RnfB of *A. woodii*, RnfB of *C. aceticum* possesses six predicted Fe-S centers. In *C. aceticum*, four of the Fe-S centers are coordinated by the motif C-X2-C-X2-C-X3-C-P and the two other centers are likely to be coordinated by C_140_ and C_219_.

**Figure 2:**
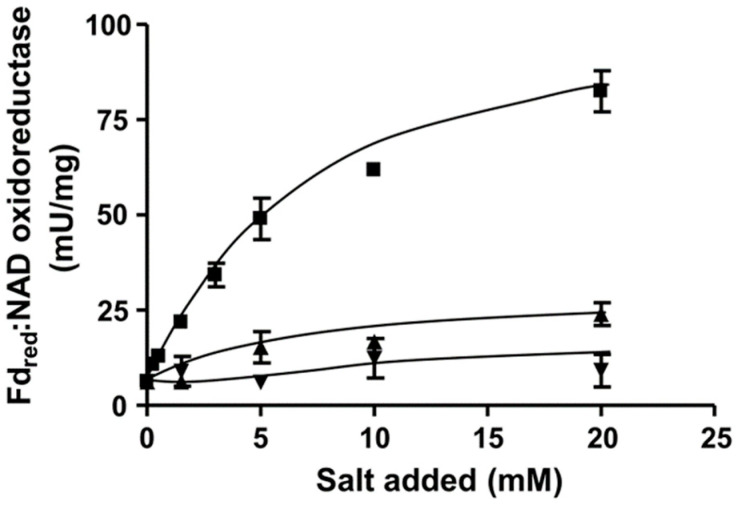
Na^+^ dependence of ferredoxin:NAD^+^ oxidoreductase (FNO) activity in *C. aceticum*. 250 μg of purified membrane were added to 1 mL buffer (20 mM Tris-HCl, 2 mM DTE, 2.2 μM resazurin, pH 7.7) and FNO was measured as described in Materials and Methods. Increasing amounts (0–20 mM) of NaCl (■), KCl (▲) or LiCl (▼) were applied to the assays. The contaminating Na^+^ concentration was 104 µM. Results show data from two replicates.

To determine whether the Rnf complex is indeed present, *C. aceticum* was grown on fructose and H_2_ + CO_2_ in medium modified after Braun et al. (1981) [16] to the late exponential phase, harvested, and the cytoplasmic membrane was prepared. In order to measure ferredoxin:NAD^+^ oxidoreductase activity we used ferredoxin isolated from *C. pasteurianum* that was reduced by CO, catalyzed by the CODH purified from *A. woodii* [17]. When membranes were incubated with CODH, ferredoxin and NAD^+^ under a CO atmosphere NAD^+^ was reduced with a rate of 85.5 ± 5.4 U/mg. NAD^+^ reduction strictly required CO, CODH, ferredoxin, membranes and NAD^+^. When no NaCl was added to the buffer, the contaminating Na^+^ concentration was only 104 µM. Under these conditions, ferredoxin-dependent NAD^+^ reduction was very low (6.3 ± 0.4 U/mg). However, upon addition of NaCl, activity was restored in a Michaelis–Menten-type fashion (Figure 2). Half maximal activity was obtained at around 5.6 mM NaCl. KCl and LiCl did not stimulate ferredoxin:NAD^+^ oxidoreductase activity (Figure 2). These data demonstrate that the Rnf complex of *C. aceticum* requires Na^+^ for activity.

### 3.2. The ATP Synthase from C. aceticum Requires Na^+^ for Activity

**Figure 3:**
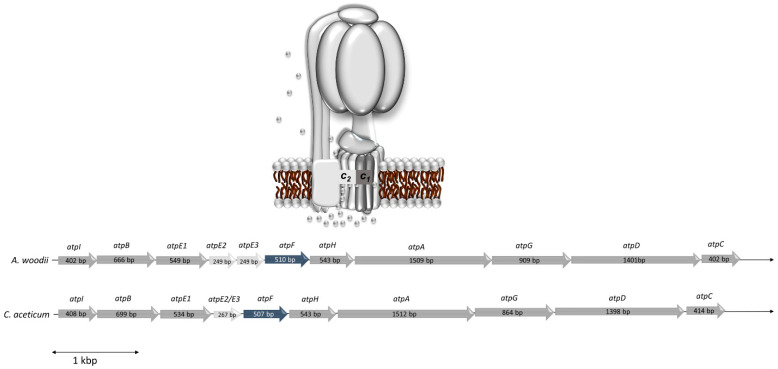
Model of the Na^+^ F_1_F_o_ ATP synthase of *C. aceticum* and comparison of the genetic organization of the *atp* operons of *A. woodi* and *C. aceticum*. The *atp* operon of *A. woodii* and *C. aceticum* coding for the F_1_F_O_-ATP synthase consists of 11 genes in *A. woodii* (Awo_c02140-Awo_c02240) and 10 genes in *C. aceticum* (CACET_c02130-CACET_c02220). The order of the genes is identical. *C. aceticum* misses *atpE3*, coding for subunit *c*_3_, which is an exact copy of subunit *c*_2_ in *A. woodii*. AtpI is not a subunit, but an assembly factor.

**Figure 4:**
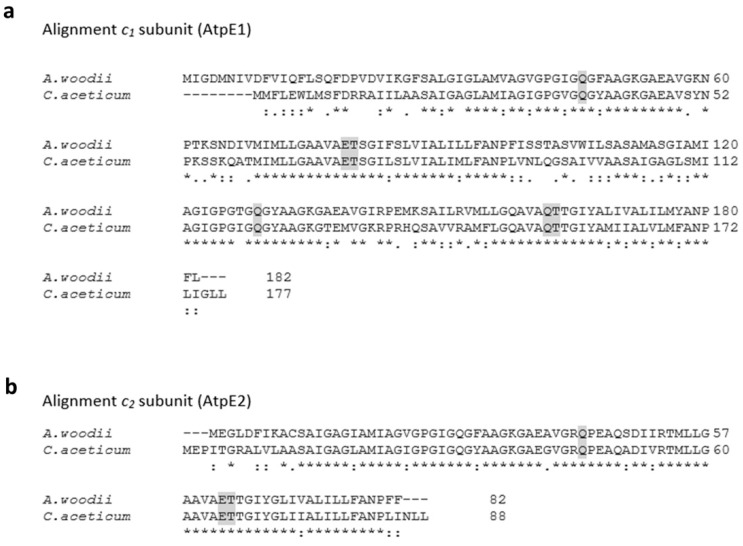
Alignment of the amino acids of subunits *c*_1_ and *c*_2_ of *A. woodii* and *C. aceticum*. Amino acids of the *c*_1_ (**a**) and *c*_2_ subunit (**b**), which are part of the *c* ring of the ATP synthase from *A. woodii* and *C. aceticum* were aligned using Clustal Omega. Highlighted in grey are the Na^+^-binding motif Q....ET of one hairpin of the *c* subunit and the second motif within a second hair pin without a functioning Na^+^-binding domain Q....QT.

The statement of Poehlein et al. (2015) [14] that the “ATPase from *C. aceticum* (encoded by CACET_c02130-CACET_c02220) does not contain an Na^+^-liganding amino acid motif”, prompted us to reexamine the *c* subunit composition of the ATP synthase from *C. aceticum* and its possible sodium ion dependence. The membrane-embedded rotor of F_1_F_O_-ATP synthases is usually made by multiple copies of one subunit, the rotor subunit *c* [26]. This subunit is membrane-integral, has two transmembrane helices and harbors the ion binding site, which is either the so-called active carboxylate (Asp or Glu) that is protonated/deprotonated in H^+^ ATPases or two more conserved residues, a glutamine in helix one and a serine/threonine in helix two, that together with the active carboxylate make the Na^+^ binding site [12,27]. The operon structure of the ATP synthases of *A. woodii* (Awo_c02140-Awo_c02240) and *C. aceticum* (CACET_c02130-CACET_c02220) is conserved (Figure 3). *C. aceticum* has two copies of genes encoding the *c* subunit of the ATP synthase (*atpE1* and *atpE2*, annotated as CACET_c02150 and CACET_c02160, respectively)—*atpE1* encodes subunit *c*_1_ and *atpE2* encodes subunit *c*_2_. Like in *A. woodii*, subunit *c*_1_ arose by duplication of an ancestral gene giving rise to a protein with four transmembrane helices [12] (Figure 4a). Hair pin one and hair pin two are 58% identical on the amino acid level. Interestingly, like in *A. woodii* [28], the first hair pin has the conserved Na^+^ binding site “Q....ET” but in the second hair pin, the gene duplication event resulted in the loss of the active carboxylate, rendering the resulting protein unable to bind either protons or sodium ions. Subunit *c*_1_ is, therefore, similar to the *c* subunit of eukaryotic ATPases that share the same feature [29,30]. Like in *A. woodii* but unlike most bacterial ATP synthases, there is a second gene encoding a *c* subunit (CACET_c02160). Subunit *c*_2_ is a “typical” bacterial *c* subunit with two transmembrane helices, i.e., one hairpin. This subunit has the conserved Na^+^-binding motif “Q....ET” (Figure 4b). *A. woodii* has a third gene, *atpE3*, that encodes a protein identical to subunit *c*_2_ [31]; this gene is missing in *C. aceticum*.

**Figure 5:**
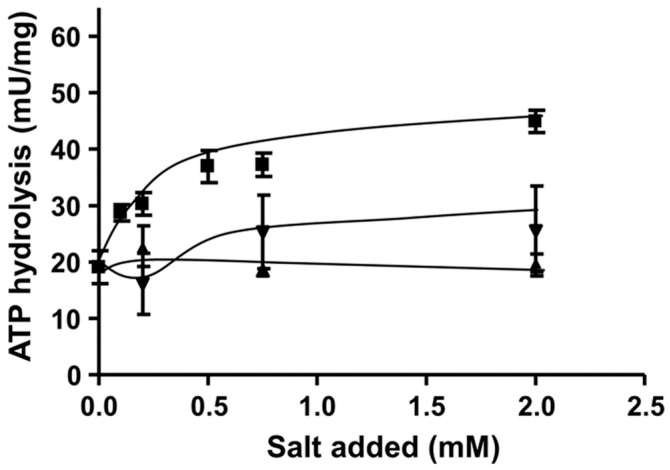
Sodium ion dependence of ATP hydrolysis activity in *C. aceticum*. 135 μg of purified membranes were added to 1200 μL ATPase buffer (100 mM Tris-HCl, 100 mM malic acid, pH 7.4) containing NaCl (■), KCl (▲) or LiCl (▼). The sample was incubated for 3 min at 30 °C. The reaction was started by adding 3 mM Tris-ATP. The contaminating Na^+^ concentration was 10 µM. Results show data from two replicates.

The presence of a sodium ion binding site prompted us to examine the effect of Na^+^ on ATP hydrolysis. Therefore, membranes were prepared as described above and assayed for ATP hydrolysis. As can be seen from Figure 5, ATPase activity was already 20 mU/µg in the presence of contaminating Na^+^ concentrations. This is in contrast to the Na^+^-Rnf. For the latter, there are few data available with respect to the ion dependence and so far, it has not been addressed whether a Na^+^-Rnf can translocate H^+^ in the absence of Na^+^. This is different in ATP synthases. Every Na^+^ ATP synthase examined to date can translocate H^+^ [32,33]. However, since at physiological conditions the Na^+^ concentration is much higher (mM range) than the H^+^ concentration (10^−4^ mM at pH 7.0), the coupling ion under physiological conditions is Na^+^ [33]. Activity of the enzymes is not strictly dependent on Na^+^ (depending on the affinities of these enzymes to Na^+^ and H^+^, which is different in different enzymes) but stimulated by Na^+^. This is also observed for ATP hydrolysis here. Activity was not stimulated by KCl but by NaCl to around 200%. The dependence of ATP hydrolysis on NaCl followed a Michaelis–Menten kinetic with half maximal activity at around 0.06 mM NaCl. The Na^+^ ATP synthases can also translocate Li^+^ but have a weaker affinity to Li^+^ [32]. The same is observed here—Li^+^ also stimulated, but stimulation was reduced.

### 3.3. The Methylene-THF Reductase Is of the MetF/MetV-Type

**Figure 6:**
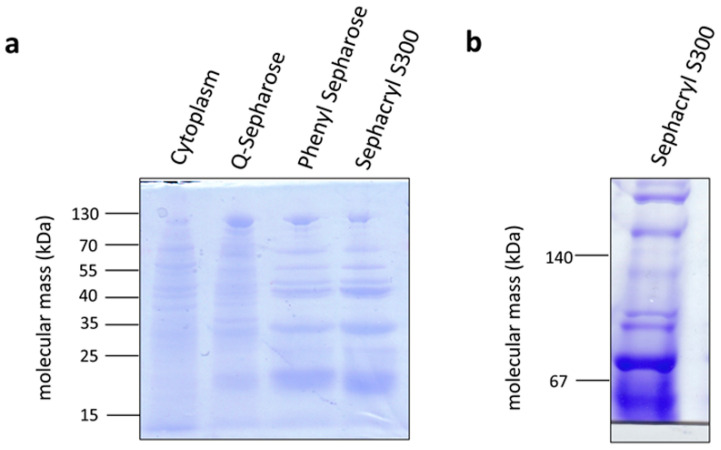
Enrichment of the methylene-THF reductase from *C. aceticum*. Samples (10 μg protein) of the cytoplasm and the purification fractions of the Q-sepharose, phenyl sepharose, and the sephacryl S300 column were separated via SDS PAGE (**a**). 10 µg of pooled active fractions of the sephacryl S300 column were also separated on a native PAGE (**b**). Gels were stained with Coomassie Brilliant Blue.

The methylene-THF reductase catalyzes the most exergonic reaction of the pathway and was, therefore, suggested some 40 years ago to be involved in energy conservation [34]. Although it was speculated some time ago that the methylene-THF reductase is the acceptor of a membrane-bound electron transport chain [35], this hypothesis has been clearly excluded by experimental data for the model acetogens analyzed [21]. However, the methylene-THF reductase may be a site for electron-bifurcation with ferredoxin as electron acceptor [36], thus providing additional fuel for the electron transport chain. This has been clearly excluded for *A. woodii* [21] but hypothesized for *Moorella thermoacetica* [37]. The *A. woodii* enzyme has the typical subunits MetF and MetV, but one additional subunit, RnfC2. The latter provides the NADH binding site and catalyzes NADH oxidation with the electron passing on to methylene-THF via MetF and MetV [21]. *M. thermoacetica* only has *metV*/*metF* genes but upstream genes are found that encode proteins with similarity to HdrC, HdrB, HdrA, and MvhD. Since these are known from electron bifurcating proteins, the idea arose that MetV/F form a complex with HdrCBA and MvhD to bifurcate electrons from NADH to methylene-THF and an unknown acceptor [37]. In contrast, *C. aceticum* has only the *metF/V* genes. To analyze the subunit composition and function of the methylene-THF reductase from *C. aceticum*, it was enriched by three consecutive chromatography steps from a cell-free extract of *C. aceticum* grown on fructose. As can be seen in Figure 6a, MetF and MetV with apparent molecular masses of 23 and 32 kDa were clearly visible. In addition, some minor contaminations were also visible. In a native PAGE, complexes of around 55 and around 75 kDa were visible indicating a stoichiometry of MetF and MetV of 1:1 (Figure 6b) or 2:1. The determination of the exact stoichiometry requires additional experiments and, most important, a purified enzyme. Interaction of MetF/MetV with EtfAB or other potential bifurcating subunits was not observed. The enriched MetF/MetV preparation used neither NADH nor NADPH as electron donor for methylene-THF reduction, even in the presence of ferredoxin. The only activity that could be determined was methylene-THF-dependent methylviologen oxidation (around 400 U/mg). 

### 3.4. The Methylene-THF Dehydrogenase Is NAD Dependent

Another important question for the overall bioenergetics is whether the methylene-THF dehydrogenase is NAD- or NADP-specific. To address this question, cells were grown on fructose and H_2_ + CO_2_, harvested in late exponential growth phase and a cell-free extract was prepared. This extract catalyzed methylene-THF oxidation with NAD^+^ (6.3 ± 1 U/mg) but not with NADP^+^ (data not shown) as electron acceptor.

### 3.5. C. aceticum Has an Electron-Bifurcating Formate Dehydrogenase

**Figure 7:**
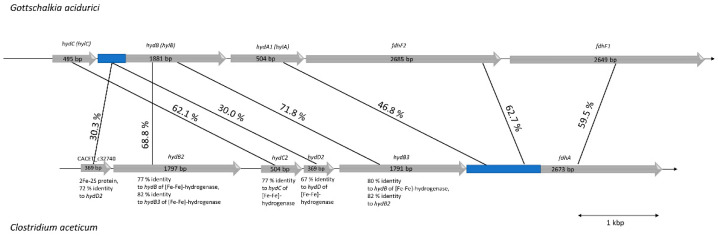
Genetic organization of the potential formate dehydrogenase (*fdh*) gene cluster in *C. aceticum* compared to the *fdh* gene cluster of *G. acidurici*. Genes coding for a potential electron-bifurcating formate dehydrogenase in *C. aceticum* are organized in a cluster consisting of six genes, whereas in *G. acidurici* there are only five [39]. The first one is CACET_c32740 coding for a 2Fe-2S protein (HydD3), which has 72% similarity to HydD2, another protein encoded in the *fdh* operon (CACET_c32710). Both genes are not found in the *fdh* gene cluster of *G. acidurici*, but they are both similar (around 30%) to the first 180 amino acids of HylB (encoded by Curi_c29400). HydB2 (encoded by CACET_c32730) and HydB3 (encoded by CACET_c32700) in *C. aceticum* share 82% identity and are both similar to HylB of *G. acidurici*. HydC2 (encoded by CACET_c32720) of *C. aceticum* is 62% similar to HylC of *G. acidurici* (Curi_c29410). In contrast to *G. acidurici*, which has two *fdhF* genes (Curi_29370 and Curi_29380), *C. aceticum* possesses only one gene (CACET_c32690) coding for a formate dehydrogenase (FdhA), which is around 60% homologous to both FdhFs of *G. acidurici*. The *fdh* gene cluster of *C. aceticum* is flanked upstream by CACET_c32750, which has similarity with a HPr kinase (a phosphocarrier protein of the phosphoenolpyruvate-dependent sugar phosphotransferase system) and downstream by CACET_c32680, which has similarity with a rubredoxin.

As suggested by Poehlein et al. (2015), *C. aceticum* contains two potential genes coding for formate dehydrogenases: CACET_c32690 and CACET_c07250. The latter is potentially misannotated. The genetic organization and similarity analyses suggests that it is homologous to a subunit of the recently described novel NADH-dependent NADPH:ferredoxin oxidoreductase (Stn) from *Sporomusa ovata* (SOV_1c07740-SOV_1c07760) [38] that is also present in *C. aceticum* (CACET_c07230-CACET_c07250). The operon containing CACET_c32690 (*fdhA*) encodes a selenocysteine-containing formate dehydrogenase with 44% homology to FdhF1/2 from *A. woodii*. Unlike *A. woodii* and *Gottschalkia acidurici*, *C. aceticum* has only the selenocysteine-containing formate dehydrogenase. Upstream of *fdhA* are genes encoding proteins with similarity to subunits of the electron-bifurcating formate dehydrogenase from *G. acidurici*. FdhA, HdB, and HydC are highly similar to the homologous protein from *G. acidurici* (with 60%–70% identity) [39]. However, compared to *G. acidurici*, *C. aceticum* does not possess a gene coding for HylA but HylA has similarity (47%) to the N-terminus of FdhA of *C. aceticum.* Interestingly, genes encoding HydB and HydD occur twice in the operon of *C. aceticum* (Figure 7). The overall similarity of the genetic organization and the gene products suggests that *C. aceticum* has an electron-bifurcating formate dehydrogenase complex that, like *G. acidurici*, catalyzes the reduction of formate with simultaneous oxidation of reduced ferredoxin and NADH.

## 4. Discussion

The data presented here demonstrate that *C. aceticum* has a sodium ion-dependent respiratory chain with a Na^+^-Rnf and a Na^+^-F_1_F_o_-ATP synthase. The latter has, like the enzyme from *A. woodii* [29], a typical V-type ATPase *c* subunit (*c*_1_) and thus, like *A. woodii*, a reduced Na^+^ to ATP stoichiometry, which is seen as an adaptation to low-energy environments [40,41,42]. Unlike *A. woodii*, which has two identical F-type ATP synthase *c* subunits [31], *C. aceticum* has only one. For the future, it would be interesting to determine the number of the different subunits in the *c* ring.

**Figure 8:**
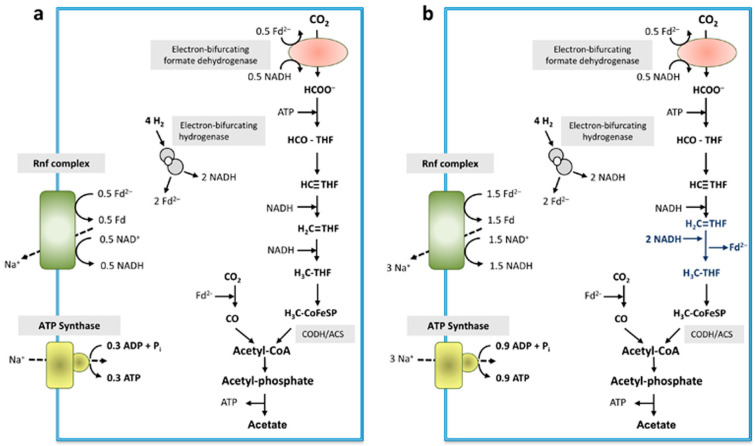
Schematic overview of energy conservation during acetogenesis from H_2_ + CO_2_ in *C. aceticum*. The electron-bifurcating hydrogenase converted 4 mol of H_2_ to 2 mol reduced ferredoxin and 2 mol NADH. Then, 0.5 mol of reduced ferredoxin together with 0.5 mol NADH are used by the electron-bifurcating formate dehydrogenase to reduce 1 mol of CO_2_ to formate. Formate gets further reduced to methyl-THF via several reduction steps: if the methylene-THF reductase uses only NADH as reductant (**a**), 2 mol NADH are required to reduce methenyl-THF to methyl-THF, whereas 3 mol NADH would be required if the methylene-THF reductase is electron bifurcating (**b**), which leads to the reduction of another mol of ferredoxin. After condensation of methyl-THF and 1 mol CO_2_ by the CODH/ACS, acetyl-CoA is further converted to acetate. For energy conservation the Rnf complex in concert with the ATP synthase are used. A Na^+^/ATP stoichiometry of 3.3 as in *A. woodii* [43] is assumed. Depending on the mode of operation of the methylene-THF reductase, either 0.3 or 0.9 mol ATP can be generated. Fd, ferredoxin; THF, tetrahydrofolate; CoFeSP, corrinoid iron-sulfur protein.

The methylene-THF reductase has only two subunits, MetF and MetV, as evident after enrichment of the enzyme and as suggested from the genomic organization. NADH was not used as electron donor, neither was NADPH. As in every other case of MetF/MetV-type methylene-THF reductases known to date, the physiological electron donor is unknown and it remains elusive whether or not the enzyme uses a second, low-potential electron acceptor that is reduced by electron bifurcation. Loss of loosely attached subunits cannot be excluded but is unlikely since also the cell-free extract did not catalyze NAD(P)H-dependent methylene-THF reduction. For our model, we assume electron bifurcation with an unknown low-potential electron acceptor. The formate dehydrogenase is suggested to be electron-bifurcating, like the homologous enzyme from *G. acidurici* [39] with NADH and reduced ferredoxin as reductant. An electron-bifurcating FeFe-hydrogenase as in *A. woodii* was described before in *C. aceticum* [14]. In sum, the data presented allowed us to depict a model for the biochemistry and bioenergetics of acetogenesis from H_2_ + CO_2_ in *C. aceticum*. It should be noted, that, in contrast to other acetogens, electron bifurcation in the course of methylene-THF reduction is not mandatory. Even without an electron-bifurcating methylene-THF reductase, the redox balance is even and under these conditions, 0.3 mol of ATP are synthesized per mol of acetate (Figure 8a) (assuming the same Na^+^/ATP stoichiometry for the ATP synthase as in *A. woodii*).

However, the redox balance is also even if an electron-bifurcating methylene-THF reductase is assumed (Figure 8b). In this model, the ATP gain is increased by 200% to 0.9 ATP/acetate.

## Data Availability

The data presented in this study are available on request from the corresponding author.
